# Epidemiologic Study of *Blastocystis* Infection in an Urban Community in the Philippines

**DOI:** 10.1155/2015/894297

**Published:** 2015-05-13

**Authors:** Maria Luz B. Belleza, Jessa Louise C. Cadacio, Maridel P. Borja, Juan Antonio A. Solon, Mildred A. Padilla, Pilarita N. Tongol-Rivera, Windell L. Rivera

**Affiliations:** ^1^Department of Parasitology, College of Public Health, University of the Philippines, Ermita, 1000 Manila, Philippines; ^2^College of Medicine, University of the Philippines, Ermita, 1000 Manila, Philippines; ^3^Department of Epidemiology and Biostatistics, College of Public Health, University of the Philippines, 1000 Manila, Philippines; ^4^Department of Veterinary Paraclinical Sciences, College of Veterinary Medicine, University of the Philippines, Los Baños, 4031 Laguna, Philippines; ^5^Institute of Biology, College of Science, University of the Philippines, Diliman, 1101 Quezon City, Philippines; ^6^Molecular Protozoology Laboratory, Natural Sciences Research Institute, University of the Philippines, Diliman, 1101 Quezon City, Philippines

## Abstract

*Blastocystis* has been considered as the most common intestinal parasite in humans and has an augmented impact on public health. However, the prevalence of this parasite in the Philippines has not been determined. To contribute to a better understanding of the epidemiology of this infection, a cross-sectional study aimed at providing the first documented data on the prevalence and correlates, sociodemographic factors, hygiene practices, source of water supply, and dog ownership, associated with *Blastocystis* infection was carried out in randomly selected communities at Pateros, Metro Manila. Fecal samples from respondents were collected and cultured in diphasic agar medium for 3–7 days and examined using light microscopy. Of the 1,271 respondents, 12.98% (95% CI: 11.13–14.83) were detected positive for *Blastocystis*. Among the correlates of *Blastocystis* infection, dog ownership was found significantly associated as confirmed by multivariate analysis. Therefore, this factor should be considered in information to create awareness about *Blastocystis* and to prevent and control *Blastocystis* infection in particular and diarrheal diseases in general. Further studies using molecular approaches to distinguish subtype and to determine genetic characteristics of isolates from humans and dogs are recommended to analyze their relationship and provide more conclusive evidence of cross-transmission.

## 1. Introduction

*Blastocystis* sp. is currently the most common intestinal protist found in human feces and is considered an emerging parasite with a worldwide distribution [1, 2]. The accepted mode of transmission of the parasite is through the fecal-oral route [3]. Its pathogenic role in humans remains uncertain since* Blastocystis* infections are both symptomatic and asymptomatic [1]. Nevertheless, it is being associated with various nonspecific gastrointestinal symptoms including diarrhea, abdominal pain, flatulence, anorexia, nausea, and vomiting [4]. The parasite may also be linked to irritable bowel syndrome and inflammatory bowel disease [5–8].

The prevalence of* Blastocystis* infection is generally higher in developing than in industrialized countries partly because of poor sanitary conditions, consumption of contaminated food or water, and close animal contact [9–12].* Blastocystis* infection is being linked with demographic factors such as age, gender, and level of education as well as exposure factors such as hygiene, source of water supply, and exposure to animals [13–16]. Moreover, higher risks of infection and high prevalence have been identified in food and animal handlers, providing conclusive evidence on its zoonotic potential [17–21]. In the Philippines, isolates of* Blastocystis* subtypes from humans were classified in the same corresponding subtypes as isolates from chickens, monkeys, and pigs in close contact with humans [22].

Diarrhea is still one of the leading causes of morbidity and is included in the top ten notifiable diseases in the Philippines [23–25]. Recent developments associating* Blastocystis* with diarrhea and showing the protist's zoonotic potential have become the bases of this community-based study. This study aimed to determine the prevalence of* Blastocystis* infection in humans using culture and light microscopy techniques and to identify which factors are associated with* Blastocystis* infection using univariate and multivariate analyses. The independent variables studied were age, gender, level of education, hygiene practices, water supply, and dog ownership.

## 2. Methods

### 2.1. Study Area and Population

**Figure 1:**
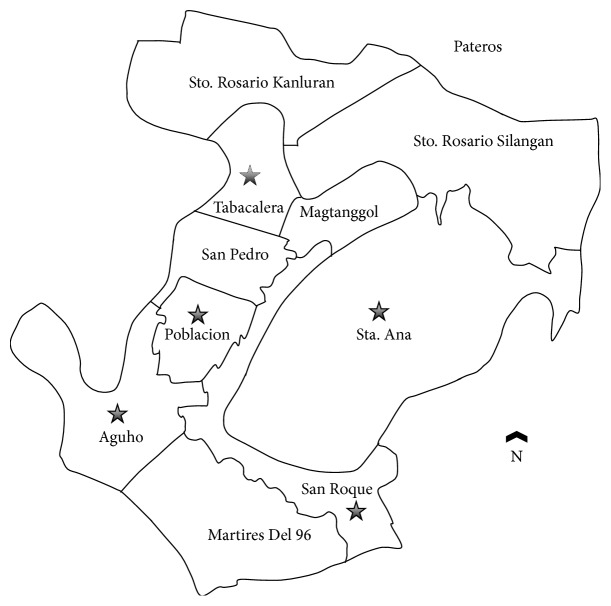
Map showing the location of the villages in Pateros, Metro Manila, Philippines, involved in the study.

A cross-sectional study was conducted in Pateros, Metro Manila, Philippines, from April 2011 to February 2012 (Figure 1). This urban community, which is mainly residential, consists of ten villages (locally called* barangay*) with a total population of 60,688 and is located southeast of Metropolitan Manila [25]. Pateros has a median age of 24 years and a sex ratio of 97.5 males for every 100 females [25]. It has population proportion of young dependents (0 to 14 years) of 32.5% and has 64.1% of economically active population (15 to 64 years). One of the great concerns of local health officials is environmental sanitation, particularly potability of water sources, toilet and excreta disposal facilities, and environmental pollution. Diarrhea was reported among the top ten notifiable diseases and was cause of consultations in the health centers in Pateros [26]. The study employed a three-stage random sampling among permanent residents of households in five different villages. The study population consisted of individuals with age > 1 year and those who had not taken antiprotozoal or antidiarrheal medications two weeks prior to sample collection.

### 2.2. Data Collection

The study utilized pretested interview schedule and direct observation to collect information on the correlates of* Blastocystis* infection such as sociodemographic factors (age, gender, and level of education) and exposure factors (hand washing, excreta disposal, source of water supply, and dog ownership). Applicability of the questionnaire was determined using a pretest group with similar characteristics as the target population. For children who have reduced ability to judge, their parents or guardians responded on their behalf.

### 2.3. Sample Collection and Processing of Fecal Specimens

Containers prelabeled with individual's name and identification number and sticks were distributed to each participant. A total of 1,271 stool specimens from humans were collected using three-stage random sampling design (municipality,* barangay* or village, and then household) and transported immediately to the Molecular Protozoology Laboratory, Natural Sciences Research Institute, University of the Philippines, Diliman, Quezon City, Philippines, for processing and examination. The presence of parasite and stool consistency (formed or diarrheic, mucoid or watery) was determined using gross examination.

### 2.4. Isolation and* In Vitro* Cultivation of* Blastocystis*

Approximately 50 mg stool samples were aseptically inoculated in diphasic medium (1.5% nonnutrient agar overlaid with buffer solution containing 137 mM NaCl, 19.6 mM Na_2_HPO_4_, 1.98 mM KH_2_PO_4_, and 3.78 mM L-asparagine) supplemented with 10% heat-inactivated horse serum (Gibco, Life Technologies, Carlsbad, CA, USA) and penicillin-streptomycin antibiotics [18]. Samples were incubated at 37°C for 3–7 days and examined for* Blastocystis* using light microscopy. Samples having the characteristic morphology of* Blastocystis* under unstained microscopic examination were considered positive for culture. Cultures were reported as negative when there was no observed parasite growth until the last day of incubation.

### 2.5. Data Management and Statistical Analysis

Data collected from individuals who were positive for culture and completed the questionnaire were computed, coded, and analyzed using STATA Standard Edition version 11.0 for Windows (StataCorp, College Station, TX, USA). Prevalence of* Blastocystis* infection for humans was estimated at 95% confidence interval (95% CI). Factors having *p* value ≤ 0.25 in univariate analysis were considered as potential confounders. A percent change in estimate of odds ratio (OR) ≥ 10 was used as basis of significant confounding effect. Identification of association between the studied factors and* Blastocystis* infection was conducted using multivariate analysis.

### 2.6. Ethical Issues

Prior to data collection, the study protocol was reviewed and approved by the Ethics Committee of the College of Public Health, University of the Philippines, Ermita, Manila, Philippines, and permission for field work was secured from the City Mayor through the Municipality Health Officer.

## 3. Results

### 3.1. Demographic and Socioeconomic Profiles

A total of 1,271 individuals aged 1 to 70 and above participated in this study with a median age of 24.7 ± 19.9 years. The majority of participants were females (60.82%) and most of them belong to age brackets 15–29 (26.12%) and 30–44 (19.35%). Overall, the studied population came from an average socioeconomic background with more than half having more than 6 years of formal education. Among the exposure factors studied (Table 1), hygiene practices showed that most of the respondents wash their hands with soap and water immediately after using toilet (79.94%). Regarding excreta disposal, seven out of 10 (78.99%) had family owned toilets. Lastly, only a few respondents owned dogs (12.27%).

### 3.2. Prevalence and Distribution of* Blastocystis *Infection

**Figure 2:**
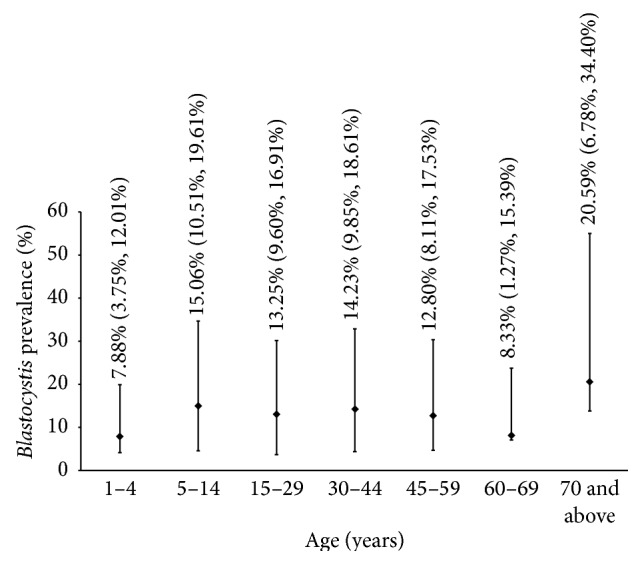
Graph showing* Blastocystis* prevalence (%) versus age (years).

The overall prevalence of* Blastocystis* infection in humans was 12.98% (95% CI: 11.13%–14.83%). Both males and females had almost equal prevalence (12.65%, 95% CI: 9.75%–15.38% and 13.20%, 95% CI: 10.81%–15.59%).  Figure 2 shows* Blastocystis* prevalence (%) according to age of the subjects in years. High school educational level on the other hand had the highest prevalence when grouped according to education (15.78%) (Table 1). Lastly, one for every four dog owners (25%, 95% CI: 18.18%–32.18%) was found positive with* Blastocystis*.

### 3.3. Statistical Analysis

Potential confounders (*p* ≤ 0.25) such as sociodemographic factors (age and level of education), hygiene practices (hand washing and excreta disposal), water supply, and dog ownership, identified using univariate analysis, were included in multivariate analysis. Among the tested potential confounders, there was no significant confounding effect to association between* Blastocystis* infection and the tested predictors (% change in estimate of OR ≥ 10). Results of multivariate analysis showed that only dog ownership was significantly associated with* Blastocystis* infection (Table 2). The odds of having* Blastocystis* infection were 2.6 times higher among dog owners than nondog owners (OR = 2.6, 90% CI: 1.9–3.7, *p* = 0.000). Associations of other factors such as sociodemographic factors (OR = 1.0, 90% CI: 0.7–1.3, OR = 3.0, 90% CI: 1.3–5.1, and OR = 1.8, 90% CI: 1.2–2.6), hygiene practices (OR = 1.6, 90% CI: 1.0–2.5 and OR = 1.7, 90% CI: 1.0–2.5), and water supply (OR = 1.4, 90% CI: 1.0–2.0) to* Blastocystis* infection were weak and not statistically significant.

## 4. Discussion

Infection with* Blastocystis* is a common health problem in many tropical and subtropical areas of the world, especially in developing countries. In the Philippines, studies on the prevalence and correlates of* Blastocystis* sp. are not well documented. This study was the first investigation on the prevalence and epidemiology of* Blastocystis* in an urban community.

This study determined the prevalence of* Blastocystis* using culture and light microscopy techniques and its association with various factors using univariate and multivariate analyses. Culture method was preferred because of its higher sensitivity and specificity compared to direct fecal smear microscopy [27] and stool polymerase chain reaction (PCR) [28, 29]. Santos and Rivera [28] considered* in vitro* culture as the gold standard in detecting* Blastocystis* cells and reported sensitivity of the following methods: 19.4% for direct fecal smear method, 19.4% for PCR from stool, and 66.7% for PCR from* Blastocystis* culture. However,* in vitro* culture is a selective technique; it is affected by the composition of medium used and the protocol applied in cultivation [28]. Thus, some isolates of* Blastocystis* are refractory to* in vitro* culture. Stensvold et al. [30] reported 100% sensitivity and specificity for culture when compared with formol-ethyl acetate concentration (FECT), trichrome staining, and xenic* in vitro* culture using PCR. Roberts et al. [31] observed 82.6% sensitivity and 100% specificity for culture. In the same year, conventional polymerase chain reaction (PCR) was found to be the most effective [31]. However, factors like requirement for special equipment (PCR machine), high cost, and need for intensive labor limited its use in this study. Compared with PCR, culture method is a cost-effective method for* Blastocystis* detection in stool, and it can also yield valid prevalence estimates. Lastly, culture method has high detection rate, since* Blastocystis* are allowed to grow and propagate, even starting with low infection.

Results show prevalence of 12.98% (~13%) in the study population. High prevalence rates were found among individuals aged 5–59 (79.61%) and those who owned dogs (25%). With regard to age, a possible reason may be an increased exposure of individuals to the parasite. Studies in endemic areas of Nicaragua [32], Bangladesh [33], and Brazil [34] showed peak prevalence of* Blastocystis* in age group 5–14. The determined prevalence rate in this study was close to the 12% reported prevalence of the Department of Parasitology in University of the Philippines Manila among prescreened clients referred from hospitals and travel agencies (unpublished data). This study shows that stool culture method is more sensitive because it allows even few* Blastocystis* to grow and multiply.

Several factors that may be associated with risk of* Blastocystis* infection, namely, sociodemographic factors (age, gender, and level of education), hygiene practices (hand washing and excreta disposal), source of water supply, and dog ownership, were analyzed in this study. Multivariate analysis identified significant association of dog ownership with* Blastocystis* infection. Dog ownership as a potential risk factor may be attributed to the zoonotic potential of* Blastocystis*. Doyle et al. [35] observed that individuals who had close contact with animals, mainly pets, could be found positive for* Blastocystis* infection. In another study, Salim et al. [20] observed animal handlers in Malaysia and reported that exposure of animal handlers to their animals was associated with* Blastocystis* infection (*p* = 0.0000313).

## 5. Conclusion

The prevalence of* Blastocystis* was 12.98% in Pateros, Metro Manila. Such data is indicative of the probability of acquiring this parasite in this community. Among the correlates studied, dog ownership was significantly associated with* Blastocystis* infection. The borderline confidence interval of this factor showed positive direction to association with* Blastocystis* infection. This factor could be considered to have important role in the transmission of* Blastocystis* infection, and understanding it provides better interventions in its prevention and control.

## Figures and Tables

**Table 1:** Prevalence of *Blastocystis *infection among permanent residents of Pateros, Philippines, according to sociodemographic and exposure factors.

	*n* (%)	% infected	95% CI
Overall	1271	12.98	11.13–14.83

*Sociodemographic factors *			
Gender			
Male	498 (39.18)	12.65	9.75–15.38
Female	773 (60.18)	13.20	10.81–15.59
Age (in years)			
1–4	165 (12.98)	7.88	3.75–12.01
5–14	239 (18.80)	15.06	10.51–19.61
15–29	332 (26.12)	13.25	9.60–16.91
30–44	246 (19.35)	14.23	9.85–18.61
45–59	195 (15.34)	12.8	8.11–17.53
60–69	60 (4.72)	8.33	1.27–15.39
70 and above	34 (2.68)	20.59	6.78–34.40
Level of education			
College	331 (26.04)	10.27	6.99–13.55
High school	431 (33.91)	15.78	12.33–19.23
Elementary	306 (24.08)	12.09	8.43–15.75
No education	203 (15.97)	12.81	8.19–17.41

*Exposure factors *			
Hygiene practices			
Hand washing			
Wash hands with soap and water immediately after using toilet	1016 (79.94)	14.17	12.03–16.32
Wash hands with water only after using toilet	162 (12.75)	8.64	4.30–12.99
Wash hands with soap and water but delays washing for more than 5 minutes after using toilet	93 (7.32)	7.52	2.13–12.92
Excreta disposal			
Family owned toilet	1003 (78.99)	14.04	11.89–16.20
Communal toilet	267 (21.01)	8.99	5.54–12.43
Water supply			
Public water system	1085 (85.37)	12.53	10.56–14.51
Communal faucet	186 (14.63)	15.59	10.35–20.82
Dog ownership			
Nondog owner	1115 (87.73)	11.30	9.43–13.16
Dog owner	156 (12.27)	25.00	18.18–32.18

*n*: number of examined.

**Table 2:** Final models for various factors and association with *Blastocystis *infection.

	Adjusted OR (90% CI)	*p* value
Final Model 1: association between sociodemographic factors and *Blastocystis *		
*Sociodemographic factors *		
Gender		
Male^∗^	1.0 (—)	—
Female	1.0 (0.7–1.3)	0.919
Age (in years)		
1–4^∗^	1.0 (—)	—
5–59	3.0 (1.3–5.1)	0.020
60 and above	2.0 (0.8–4.5)	0.186
Level of education		
College^∗^	1.0 (—)	—
High school	1.8 (1.2–2.6)	0.179
Elementary	1.4 (0.9–2.1)	0.040
No education	3.0 (1.7–5.4)	0.002

Final Model 2: association between hygiene practices and *Blastocystis *		
*Hygiene practices *		
Hand washing		
With soap and water immediately^∗^	1.0 (—)	—
With water only or delayed washing	1.6 (1.0–2.5)	0.091
Excreta disposal		
Family owned toilet^∗^	1.0 (—)	—
Communal faucet	1.7 (1.1–2.5)	0.036

Final Model 3: association between source of water supply and *Blastocystis* infection		
*Water supply *		
Public water system^∗^	1.0 (—)	—
Communal toilet	1.4 (1.0–2.0)	0.135

Final Model 4: association between dog ownership and *Blastocystis* infection		
*Dog ownership *		
Nondog owner^∗^	1.0 (—)	—
Dog owner	2.6 (1.9–3.7)	0.000

^∗^Reference.
